# Sodium bicarbonate versus isotonic saline solution to prevent contrast-induced nephropathy: a systematic review and meta-analysis

**Published:** 2015-09-30

**Authors:** Carlos Andres Zapata-Chica, Diana Bello Marquez, Lina Maria Serna-Higuita, John Fredy Nieto-Ríos, Fabian David Casas-Arroyave, Jorge Hernando Donado-Gómez

**Affiliations:** 1 Pediatric Nephrologist. The University of Antioquia, Medellin, Colombia.; 2 Pediatric Nephrologist. Pablo Tobón Uribe Hospital, The University of Antioquia. Medellin, Colombia; 3 Nephrologist. Pablo Tobón Uribe Hospital, The University of Antioquia, Medellin, Colombia; 4 Anesthesiologist. The University of Antioquia. Medellin, Colombia; 5 Clinical Epidemiologist. Pablo Tobón Uribe Hospital, Medellin, Colombia

**Keywords:** Contrast-induced nephropathy, acute kidney injury, sodium bicarbonate, sodium chloride

## Abstract

**Introduction:**

Contrast-induced nephropathy is one of the main causes of acute kidney injury and increased hospital-acquired morbidity and mortality. The use of sodium bicarbonate for nephroprotection has emerged as a preventative strategy; however, its efficacy is controversial compared to other strategies, such as hydration using 0.9% saline solution.

**Objective:**

To compare the effectiveness of sodium bicarbonate vs. hydration using 0.9% saline solution to prevent contrast-induced acute kidney injury.

**Methods:**

A systematic review of studies registered in the COCHRANE, PUBMED, MEDLINE, LILACS, SCIELO and EMBASE databases was conducted. Randomized controlled studies that evaluated the use of 0.9% saline solution vs. sodium bicarbonate to prevent contrast-induced nephropathy were included.

**Results:**

A total of 22 studies (5,686 patients) were included. Sodium bicarbonate did not decrease the risk of contrast-induced nephropathy (RD= 0.00; 95% CI= -0.02 to 0.03; *p*= 0.83; I^2^= 0%). No significant differences were found in the demand for renal replacement therapy (RD= 0.00; 95% CI= -0.01 to 0-01; I^2^= 0%; *p*= 0.99) or in mortality (RD= -0.00; 95% CI= -0.001 to 0.001; I^2^= 0%; *p*= 0.51).

**Conclusions:**

Sodium bicarbonate administration is not superior to the use of 0.9% saline solution for preventing contrast-induced nephropathy in patients with risk factors, nor is it better at reducing mortality or the need for renal replacement therapy.

## Abstract (es)

**Introducción:**

La nefropatía inducida por medio de contraste es una de las causas principales de lesión renal aguda, lo cual incrementa la morbilidad y mortalidad intrahospitalaria. La nefroprotección con bicarbonato de sodio ha surgido como una estrategia preventiva, sin embargo su eficacia es controversial cuando se compara con estrategias como la hidratación con solución salina al 0.9%.

**Objetivo:**

Comparar la efectividad del bicarbonato de sodio versus la hidratación con solución salina al 0.9% en la prevención de la lesión renal aguda inducida por contraste.

**Métodos:**

Se realizó una revisión sistemática de los estudios registrados en COCHRANE, PUBMED, MEDLINE, LILACS, SCIELO y EMBASE. Se incluyeron estudios aleatorizados, controlados donde se evaluó el uso de solución salina al 0.9% versus bicarbonato de sodio para prevenir la nefropatía por medio de contraste.

**Resultados:**

Se incluyeron 22 estudios (5,686 pacientes). El bicarbonato de sodio no disminuyó el riesgo de nefropatía inducida por contraste (DR= 0.00 IC95%= -0.02 -0.03; *p*= 0.83, I^2^= 0%). Tampoco se encontró diferencia significativa en la necesidad de terapia de reemplazo renal (DR= 0.00 IC 95%= -0.01-0.01, I^2^= 0%, *p*= 0.99); ni en la mortalidad (DR= -0.00, IC 95%= -0.001-0.001, I^2^= 0%, *p*= 0.51).

**Conclusiones:**

La administración de bicarbonato de sodio no es superior al suministro de solución salina al 0.9% en la prevención de nefropatía inducida por medio de contraste en pacientes con factores de riesgo. Su uso tampoco es superior en la reducción de mortalidad y el requerimiento de terapia de reemplazo renal.

## Introduction 

Contrast-induced nephropathy (CIN) is a usually reversible form of acute kidney injury (AKI) that occurs after the intravenous or intra-arterial administration of contrast media. Contrast-induced nephropathy is the third most common cause of *de novo *AKI among hospitalized patients; it is associated with an increased risk of complications such as acute myocardial infarction, longer hospital stays and higher costs, especially when its management requires the use of renal replacement therapy 1,2.

 Contrast-induced nephropathy is diagnosed according to some of the following criteria: a) an absolute increase in serum creatinine of >0.5 mg/dL, b) a relative increase in serum creatinine of >25% with respect to baseline or c) an estimated glomerular filtration rate (GFR) of less than 30-60 mL/min/1.73 calculated using the recommended equations within the first 24 to 72 h after exposure to contrast media in the absence of an alternative explanation for the impairment 3,4. Other definitions published in the literature include a serum creatinine increase of ≥0.3 mg/dL or to 1.5 times baseline within the previous 7 days or a urine volume of <0.5 mL/kg/h for 6 hs after exposure 5; however, the first two definitions are currently supported by the highest consensus. 

The exact pathogenesis of CIN is uncertain. It has been postulated that hypoxic injury and the generation of free radicals induced by exposure to contrast media plays an important role 6. At present, prevention measures are the best option for all patients at risk of developing CIN, and different preventive strategies have been proposed, including periprocedural hydration with 0.9% Normal saline solution (NSS) 7,8 and the administration of sodium bicarbonate (SB) 9,10. These therapies appear to have a protective effect against CIN; however, the results of multiple trials that have used these measures have been controversial and have not clarified the best management strategy 11. Various systematic reviews and meta analyses have shown that SB was beneficial for preventing CIN but did not improve other clinical outcomes, such as death, heart failure and the need for renal replacement therapy (RRT); additionally, these meta-analyses also showed publication bias and significant heterogeneity 11,12. The aim of this study was to determine the effectiveness of SB compared to 0.9% NSS for preventing CIN in patients older than 18 yrs who were exposed to contrast media.

##  Materials and Methods

###  Protocol

This review and meta-analysis was performed according to the Cochrane Collaboration 13 and PRISMA-P 14 guidelines for the development of systematic review protocols.

###  Eligibility criteria

This review included controlled clinical trials that compared SB infusion to 0.9% NSS as a prevention strategy for CIN among adults who were older than 18 yrs and had risk factors for kidney disease or a diagnosis of chronic kidney disease or had undergone coronary procedures, interventional radiology or diagnostic tests that required contrast media. Studies published in the English- or Spanish-language literature or databases were included, with no restriction placed on the time of publication.

 Contrast-induced nephropathy is defined as a glomerular filtration rate (GFR) decrease greater than 25% calculated using the Chronic Kidney Disease Epidemiology Collaboration (CKD-EPI) formula or as an increase in serum creatinine greater than 0.5 mg/dL compared to the baseline within 48 h of the procedure or an absolute increase of 25% compared to the baseline 4,15. Additionally, some secondary outcomes were evaluated, including the need for renal replacement therapy (RRT), the exchange difference with basal serum creatinine, and mortality.

###  Information sources and search strategies 

A search of studies recorded since the formation of The Renal Group of the Cochrane Collaboration using the term "contrast-induced nephropathy" was conducted. Additionally, all of the clinical trials registered in the Cochrane Central Register of Controlled Trials (CENTRAL) were searched using the terms Nephropathy, Bicarbonate, Saline Solution, and Contrast Media (the search strategy is detailed in the annexes). Various electronic databases were also searched using terms and highly sensitive strategies to identify controlled trials. For PUBMED, the following terms were employed: "contrast nephropathy", "sodium bicarbonate", "sodium chloride" and "renal failure"; for EMBASE, "sodium chloride**", **"acute renal failure", "contrast nephropathy", and "sodium bicarbonate" were used. Additionally, the Latin American databases LILACS and SCIELO were searched using terms "*nefropatía inducida por medio de contraste*", "*bicarbonato de sodio*", and "*solución salina*".

###  Article selection

The titles and summaries of the studies identified by the search were independently evaluated by two authors (CAZ and DB), and the full studies were examined for their potential to meet the eligibility criteria. A third author (LMS) resolved any disagreement between the two evaluating authors. After the analysis, the authors decided which studies fulfilled the inclusion criteria. The agreement among the evaluators was assessed using the Kappa formula. 

###  Data extraction

One author (LMS) was designated to develop a standard electronic format for data collection. The other authors (CAZ and DB) evaluated and approved the format prior to data extraction; however, LMS performed double data entry to correct errors and missing data. 

The following information was extracted from each study: age, reason for exposure to contrast media, diagnosis, history of kidney and/or diabetes, doses and types of contrast media used, type of intervention performed (SB dose, time before treatment); control (doses and duration of infusion); and outcomes measured (CIN, need for RRT and death).

###  Analysis

**Risk of bias. **To determine the risk of bias, the format proposed by the Cochrane Collaboration for assessing the risk of bias in primary studies was used 13. For each study, the authors determined whether the subjects and treatments were randomized, how the randomization sequence was concealed, who in the study was blinded to the intervention and how the blinding occurred, data collection, the amount of missing data and missing data were managed, the type of analysis performed and whether a reporting bias was generated. Two evaluators (LMS and DB) performed this analysis separately, and disagreements were resolved by consensus with a third reviewer (CAZ). To determine the consensus, Kappa was used.

**Summary of the measures and analysis plan. **For each outcome and each study, a 2x2 table was generated wherein the number of patients who experienced an event or outcome in each comparison group and the total number of patients in each group were summarized. For each statistic calculated, the program Review Manager^®^ version 5.3 was applied, with the exception of the meta-regression analysis, for which the program Comprehensive Meta-Analysis^®^ 2.0 was used. The treatment weighting was calculated throughout the study. The results are presented as risk differences (RD) with their 95% confidence intervals (CI) for dichotomous variables and mean differences with their 95% confidence intervals for continuous variables. The DerSimonian and Laird random effects model was used for all outcomes. This method was chosen to generate estimates and a conservative CI because it includes the intra- and intervariance of the studies. For all of the results, two-tailed *p* values are shown, and *p *<0.05 was considered statistically significant. 

To identify the potential risk of heterogeneity, the statistical tau test^2^, with *p *<0.1 indicating statistical significance, and the I^2^ test, in which a value greater than 50% indicates heterogeneity, were applied. 

Subgroup analyses were performed based on the methodological quality standards for studies, the use of N-Acetylcysteine (NAC) and the type of contrast medium employed (iso-osmolar or hypo-osmolar), given that the risk of kidney injury is greater when hypo-osmolar contrast media are used in contrast to iso-osmolar ones. Additionally, a meta-regression was performed to evaluate whether the presence of diabetes or the quantity of contrast medium used could be related to the development of CIN. In this analysis, two-tailed *p* values were reported, and values less than 0.05 were considered statistically significant for the interaction or the regression coefficient. 

###  Publication bias throughout the study 

A funnel plot was generated to evaluate the presence of publication bias. For this purpose, the inverse variance was plotted against the logarithm of the RR. The presence of asymmetry was evaluated; however, the evaluation may be subjective. Egger's linear regression test was conducted and was weighted by evaluating the association between the study size and the estimated treatment effect. A value of *p *<0.05 was considered statistically significant for publication bias. 

## Results 

### Study selection 

**Figure 1.**
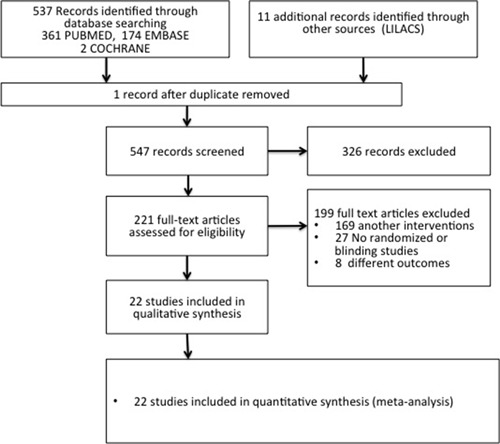
Flow chart of study selection for sodium bicarbonate and isotonic saline solution. Flowchart of the studies where the results of the search and the evaluation process are illustrated and the selection of studies for inclusion in the review.

A total of 548 reports were found during the initial search of the bibliographical databases EMBASE, PUBMED, COCHRANE, SCIELO and LILACS. After the initial assessment, 327 publications were excluded; the full text of the 221 remaining reports was analyzed. Among those, 199 studies were excluded because they examined another type of intervention, were not randomized and/or controlled or did not measure the proposed outcomes. Finally, 22 clinical randomized studies with a total of 5,686 patients in which a main outcome of CIN could be analyzed were reviewed (Fig. 1).

### Methodology 

**Figure 2.**
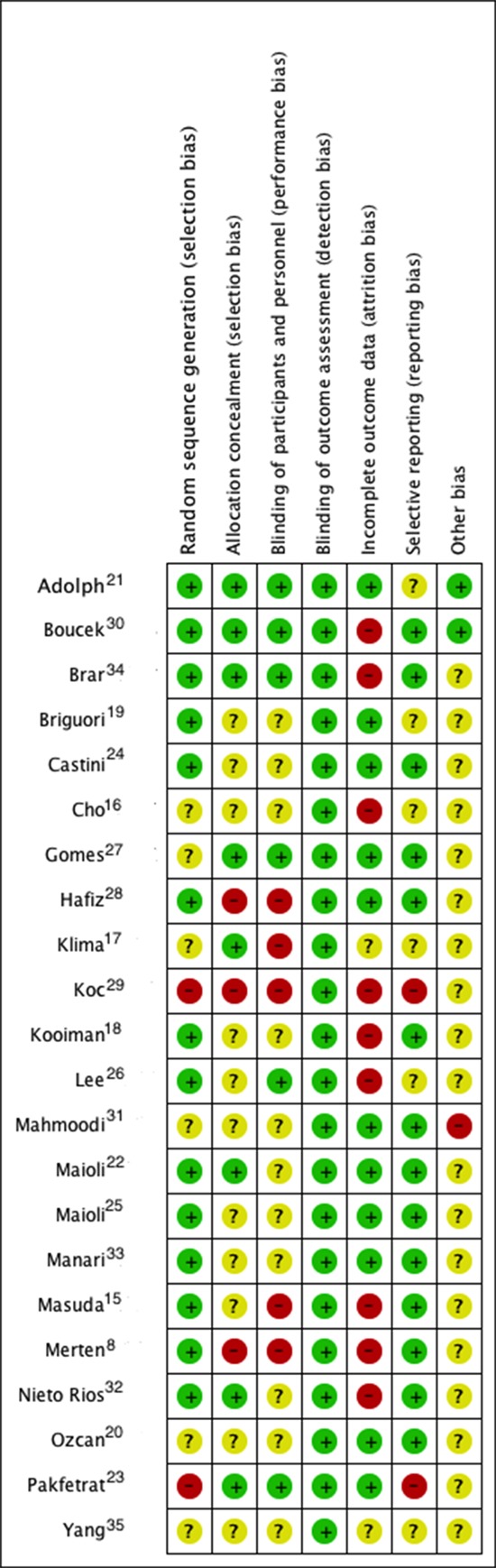
Risk of bias in the individual studies. Summary of risk of bias in the individual studies, which were grouped into seven domains that assessed the different potential sources of bias. The rating was performed by color: green indicates a low risk of bias, yellow indicates an unknown risk, and red indicates a high risk of bias.

The 22 studies selected for review comprised clinical randomized controlled studies published in English. However, only eight of the studies (36%) concealed the randomization sequence (Fig. 2).

### Participants 

The studies included a total of 5,686 patients who contributed to the primary outcomes. In all, the patients had a history of kidney disease or a high risk of developing it, which was determined using the baseline serum creatinine measurement or the GFR: In 12 of the studies, the creatinine cut-off was defined as greater than 1.1 mg/dL to 1.5 mg/dL, and in two of the studies, the GFR cut-off was less than 60 mL/min/1.73. In 13 studies, low-osmolality contrast media was used, eight were iso-osmolar and in one study both contrast medium were used.

### Intervention

Among the studies that included SB administration, 18 administered SB diluted with 5% dextrose in distilled water (D5%DW), and 4 did not specify the dilution of bicarbonate used 16-19; however, stabilized bicarbonate is only achieved with the addition of dextrose, so it can be assumed that this dilution was performed. The quantity of D5%DW for dilution differed among the studies; in 16 studies, 154 mL of bicarbonate (1,000 mEq/L) was diluted in 846 mL of D5%DW (studies 8,15,20-32,33); in the remaining studies, different mixtures of bicarbonate and D5%DW were used; the infusion speed was 1 to 3 mL/k/h within 1 to 12 h before the radiological procedure was performed and then between 6 and 12 h post-procedure (Table 1). In six studies 20,21,23,27,29,32, NAC was used along with the sodium bicarbonate. NAC was always administered between 6 and 12 h before the intervention. All of the trials included patients who had undergone coronary procedures, a type of coronary angiography or percutaneous coronary intervention. Furthermore, four studies included patients who underwent computerized axial tomography.

**Table 1.** Studies included in meta-analysis.

Study (reference)	Age (years)	Serum creatinine or GFR	Diabetes N (%)	Procedure	Trial design	Dose of contrast media (DS)
Merten^8^	>18; NaCl 69.2 (32-87); NaHCO_3_ 66.7 (37-88)	Cr >1.1 mg/dL	NaCl: 27 (46%); NaHCO3 30 (50%)	CA. CAT	NaHCO_3_ 154 mL of 1,000 mEq/L to 846 mL of dextrose 5%. 3 mL/kg for 1 h before CM followed by an infusion of 1 mL/kg/h for 6 h after versus NaCl 154 mEq/L in 5% dextrose and H2O. 1 mL/kg for 6 h before CM followed by an infusion of 1 mL/kg/h for 12 h after	NaCl 134 mL (63); NaHCO_3_ 130 mL (72)
Masud^36^	>20; NaCl 76 (11); NaHCO_3_ 75 (8)	Cr >1.1mg/dL; GFR <60 mL/min/1.73	NaCl: 10 (35%); NaHCO3: 8 (27%)	CA. PCI	NaHCO_3_ 154 mL of 1,000 mEq/L to 846 mL of 5% Dextrose and H_2_O versus NaCl 0.9%. 3 mL/kg for 1 h before CM followed by an infusion of 1 mL/kg/h for 6 h after	NaCl 120 mL (61); NaHCO_3_ 112 mL (89)
Briguor^37^	>18; NaCl 71 (9); NaHCO_3_ 70 (9)	Cr >2 mg/dL GFR <40 mL/min/1.73	NaCl: 61 (55%); NaHCO3: 53 (49%)	CA. PA. PCI	NaHCO_3_ 154 mL of 1,000 mEq/L to 846 mL of dextrose 5% + NAC. 3 mL/kg for 1 h before CM followed by an infusion of 1 mL/kg/h for 6 h after versus NaCl 0.9%+NAC. 1 mL/kg for 12 h before and after CM	NaCl 179 mL (102); NaHCO_3_ 169 mL (92)
Ozcan^38^	>18; NaCl 70 (40-84); NaHCO_3_ 68 (46-86)	Cr >1.2 mg/dL	NaCl: 47.7%; NaHCO3: 42%	CA. PCI	NaHCO_3_ 154 mL of 1,000 mEq/L to 846 mL of dextrose 5% + NAC for 1 mL/kg for 6 h before and after CM versus NaCl 0.9%+NAC. 1 mL/kg for 6 h before and after CM	NaCl 110 mL (30-270); NaHCO_3_ 100 mL (50-300)
Adolph^39^	>18; NaCl 72.7 (6.5); NaHCO_3_ 70.1 (8.4)	Cr >1.2 mg/dL GFR <63 mL/min/1.73	NaCl: 23 (28.3%); NaHCO3: 26 (36.6%)	CA	NaHCO_3_ 154 mL of 1,000 mEq/L to 846 mL of 5% dextrose in H_2_O versus NaCl 154 mEq/L in 5% dextrose in H_2_O. 2 mL/kg/h for 2 h before CM followed by an infusion of 1 mL/kg/h for 6 h after	NaCl 138 mL (52); NaHCO_3 _141 mL (50)
Maioli^40^	>18; NaCl 74 (70-79); NaHCO_3_ 74 (67-79)	Cr >1.5 mg/dL GFR <60 mL/min/1.73	NaCl: 59 (23%); NaHCO3: 62 (25%)	CA. PCI	NaHCO_3_ 154 mL of 1,000 mEq/L to 846 mL of 5% dextrose and H_2_O + NAC 3 mL/kg for 1 h before CM followed by an infusion of 1 mL/kg/h for 6 h after versus NaCl 0.9% + NAC 1 mL/kg for 12 h before and after CM	NaCl 167 mL (66); NaHCO_3_ 171 mL (69)
Brar^41^	>18; NaCl 71 (65-76); NaHCO_3_ 71 (65-75)	GFR <60 mL/min/1.73	NaCl: 81 (45.5%); NaHCO3: 76 43.4%)	CA	NaHCO_3_ 1,000 mEq/L, 150 mL versus NaCl 0.9%. 3 mL/kg for 1 h before CM followed by an infusion of 1 mL/kg/h for 4 h after	NaCl 137 mL (89-247); NaHCO_3_ 126 mL (80-214)
Pakfetrat^42^	>18; NaCl 58.4 (11.5); NaHCO_3_ 57.8 (11.2)		NaCl: 31 (32.3%); NaHCO3: 26 (27%)	CA	NaHCO_3_ 154 mL of 1,000 mEq/L to 846 mL of 5% dextrose and H_2_O. 3 mL/kg for 1 h before CM followed by an infusion of 1 mL/kg/h for 6 h after versus NaCl 0.9% 1 mL/kg for 6 h before and after	NaCl 67 mL (41.1); NaHCO3 58 mL (32.7)
Cho^22^	>18; NaCl 77.33 (9.39); NaHCO_3_ 78.47 (8.72)	Cr >1.1 mg/dL GFR <60mL/min/1.73	NaCl: 8 (29.6%); NaHCO3: 9 (42.8%)	CA	NaHCO_3_ 154 mL of 1000 mEq/L to 846 mL of 5% dextrose and H_2_O. 3 mL/kg for 1 h before CM followed by an infusion of 1 mL/kg/h for 6 h after versus NaCl 154 mEq/L. 3 mL/kg for 1 h before CM followed by an infusion of 1 mL/kg/h for 6 h after	NaCl 122.59 mL; NaHCO_3_ 136.31 mL
Castini^43^	>18; NaCl 72.7 (8.2); NaHCO_3_ 70 (8.3)	Cr >1.2 mg/dL	NaCl: 10 (20%); NaHCO3: 18 (35%)	CA. PCI	NaHCO_3_ 154 mL of 1,000 mEq/L to 846 mL of 5% dextrose and H2O. 3 mL/kg for 1 h before CM followed by an infusion of 1mL/kg/h for 6 h after versus NaCl 0.9% 1mL/kg for 12 h before and after CM	NaCl 196.4 mL (127.7); NaHCO_3_ 179.2 mL (125.1)
Lee^23^	>18; NaCl 67.5 (62-72); NaHCO_3_ 68.5 (63-73)	Cr >1.1 mg/dL GFR <60 mL/min/1.73	NaCl: 189 (100%); NaHCO3: 193 (100%)		NaHCO_3_ 154mL of 1,000 mEq/L to 846 mL of 5% dextrose and H_2_O. 3 mL/kg for 1 h before CM followed by an infusion of 1 mL/kg/h for 6 h after versus NaCl 0.9% 1 mL/kg for 12 h before and after CM	NaCl 120 mL (79-223); NaHCO_3_ 113 mL (80-220)
Maioli^24^	18; NaCl 66 (12); NaHCO_3_ (13)		NaCl:11 (20.7%); NaHCO3: 31(20.7%)	CA	NaHCO_3_ 154 mL of 1,000 mEq/L to 846 mL of 5% dextrose and H_2_O. 3 mL/kg for 1 h before CM followed by an infusion of 1mL/kg/h for 6 h after versus NaCl 0.9% 1mL/kg for 12 h before and after CM	NaCl 216 mL (101); NaHCO_3_ 208 mL (92)
Gomes^44^	>18; NaCl 64.5 (12); NaHCO_3_ 64.1 (12)	Cr >1.2 mg/dL GFR <50 mL/min/1.73	NaCl: 45 (29.8%); NaHCO3: 43 (8.7%)	CA. PCI	NaHCO_3_ 154 mL of 1,000mEq/L to 846 mL of 5% dextrose and H_2_O 3 mL/kg for 1 h before CM followed by an infusion of 1 mL/kg/h for 6 h after versus NaCl 0.9% 3 mL/kg for 1 h before CM followed by an infusion of 1 mL/kg/h for 6 h after	NaCl 125 mL (87); NaHCO_3_ 124 mL (65)
Klima^25^	>18; NaCl 75 (70-82); NaHCO_3_ 78 (70-82)	Cr >1 mg/dL GFR <60 mL/min/1.73	NaCl: 30 (34%); NaHCO3: 34 (39%)	CAT. CA. PA. PCI	NaHCO_3_ 166 mEq/L, 3 mL/kg for 1 h before CM followed by an infusion of 1 mL/kg/h for 6 h after versus NaCl 0.9% 1 mL/kg for 8 h before CM followed by an infusion of 1 mL/kg/h for 12 h after	NaCl 100 mL (80-163); NaHCO_3_ 100 mL (80-143)
Hafiz^45^	>18; NaCl 73 (63-80); NaHCO_3_ 74 (65-80)	Cr >1.4 mg/dL GFR <60 mL/min/1.73	NaCl: 73 (45.3%); NaHCO3: 78 (49.1%)	CA	NaHCO_3_ 154 mL of 1,000 mEq/L to 846 mL of 5% dextrose and H_2_O + NAC 3 mL/kg for 1 h before CM followed by an infusion of 1 mL/kg/h for 6 h after versus NaCl 0.9%+ NAC 1 mL/kg for 12 h before and after CM	NaCl 100 mL (80-140); NaHCO_3_ 110 mL (75-155)
Koc^46^	>18; NaCl 62 (9); NaHCO_3_ 62 (9)		NaCl: 100%; NaHCO3: 100%	CA	NaHCO_3_ 154 mL of 1,000 mEq/L to 846 mL of 5% dextrose and H_2_O + NAC 3 mL/kg for 1 h before CM followed by an infusion of 1 mL/kg/h for 6 h after versus NaCl 0.9%+ NAC 1 mL/kg for 12 h before and after CM	NaCl 90 mL (85-100); NaHCO_3_ 90 mL (90-100)
Boucek^47^	>18; NaCl 67 (10); NaHCO_3_ 63 (11)	Cr >1.1 mg/dL	NaCl: 59 (100 %); NaHCO3: 61 (100%)	CA	NaHCO_3_ 154mL of 1,000 mEq/L to 846 mL of 5% dextrose and H_2_O versus NaCl 5.85% 154 mL + 846 mL in 5% dextrose and H_2_O. 3 mL/kg for 1 h before CM followed for 1 mL/k/h for 6 h after	NaCl 104 mL (32); NaHCO_3_ 115 mL (47)
Kooiamn^48^	>18; NaCl 72.5 (9.5); NaHCO_3_71.6 (9.8)	GFR <60 mL/min/1.73	NaCl: 76 (27%); NaHCO3: 71 (26.6%)	CAT. CA	NaHCO_3_ 1.4% 250 mL IV versus NaCl 1,000 mL before and after CM	NaCl 104.7 mL (21.6); NaHCO_3_ 105.7 mL (21)
Mahmoodi^49^	>18; NaCl 64.4 (11.07); NaHCO_3_ 64.96 (10.29)		No date	CA	NaHCO_3_ 154 mL of 1,000 mEq/L to 846 mL of 5% dextrose and H_2_O + NAC versus NaCl 0.9%+NAC 3 mL/kg for 6 h before and after	No date
Nieto-Rios^50^	>18; NaCl 59.8 (17.2); NaHCO_3_ 60.7 (17.1)	Cr >1.2 mg/dL	NaCl: 39 (34.5%); NaHCO3: 43 (40.2%)	CAT. CA. PCI	NaHCO_3_ 75 mL of 1,000 mEq/L to 425 mL of 5% dextrose and H_2_O. 3 mL/kg for 1 h before CM followed by an infusion of 1 mL/kg/h for 6 h after versus NaCl 0.9% 1 mL/kg for 6 h before and after	NaCl 100.6 mL (38.2); NaHCO_3_ 99.3 mL (43.9)
Manari^51^	>18; NaCl 65 (12.4); NaHCO_3 _63.9 (12.9)	No date	NaCl: 49 (16.7%); NaHCO3: 49 (16.4%)	CA. PCI	NaHCO_3 _77 mL 433 mL of 5% dextrose and H_2_O. 1 mL/kg for 12 h before CM followed by an infusion of 1 mL/kg/h for 12 h after versus NaCl 0.9% 1 mL/kg for 12 h before and after	NaCl 199 mL (77); NaHCO_3_ 194 mL (83)
Yang^52^	>18; NaCl 59.6 (11.08); NaHCO_3_ 58.71 (10.9)	No date	NaCl: 37 (22.9%); NaHCO3: 27 (16.9%)	CA. PCI	NaHCO_3_ 450 mL 433 mL of 1,050 of 5% dextrose and H_2_O. 1.5 mL/kg for 6 h before and after CM. versus NaCl 0.9% 1.5 mL/kg for 6 h before and after	NaCl 124 mL (63.8); NaHCO_3 _127 mL (48.09)

Cr: serum creatinine; NaCl: sodium cloruro; NaHCO3: sodium bicarbonate; NAC: N-Acetilcisteíne; CA: coronary angiography,; PCI: percutaneous coronary intervention, PA: peripheral angiography, GFR: glomerular filtration rate; CAT: computerized axial tomography; CM: contrast media.

### Control

In all of the studies, the control was performed with 0.9% NSS, usually administered between 6 and 12 h before and after the procedure. On 6 occasions, NAC was added to the treatment 20,23,27,29,32 and was administered between 6 and 12 h before intervention. 

### Primary outcomes 

 The primary outcome evaluated in 13 studies was the presence of CIN, defined as a 25% elevation in serum creatinine above the baseline or a 0.5-mg/dL increase during the first 48 hours after the contrast medium was administered 8,15,16,17,19-22,27,29-32; in seven studies, the same definition was used, but CIN was diagnosed up to 5 days post-contrast medium administration 23,25,26,28,33-35; the other two studies used the maximum increase in serum creatinine as an outcome measure 18,24. 

### Secondary effects

 The need for RRT was evaluated in 15 studies and was defined across the board as the need for hemodialysis 48-72 h after exposure to contrast media secondary to acute renal failure 18-23,28,30-34; in one study, the need for hemodialysis up to 30 days after contrast medium exposure was considered 27. In 15 studies, mortality was defined as death by any cause within 28 days post procedure 15,16-19,21-23,26-29,31,32,34. The difference in creatinine was assessed in 8 studies, which reported the mean differences in the creatinine level before and after exposure to contrast media in both groups. Table 1 shows the characteristics of each study included in the systematic review. 

### Risk of bias in the included studies 

The methodological quality details of the individual studies are presented in Figure 2. To assess the risk of bias, the studies were evaluated according to a Kappa value of 0.67.

### Randomization and concealment

The random allocation sequence was judged inappropriate in two studies (9.1%) 24,30 given that it was not specified and from reading it can be inferred that there was not any system of randomization. While randomization was effectively reported in 6 studies (27.3%), they failed to specify how they were conducted 17,18,21,28,32,35; in other studies, randomization was deemed adequate. In relation to the concealment of the random sequence, it was deemed adequate in 8 studies (36%) 15,18,22-24,28,31,34; in 11(50.0%) it was deemed uncertain given that the type of concealment was not defined and three (13.7%) of the concealments were equally not conducted 8,29,30.

### Blinding

 In 5 studies (9.1.%), the blinding of the patients and the doctors who conducted the intervention was rated adequate 22,24,28,31,34; in 12 studies (54.5%), it was unclear how the blinding was performed; and in 5 studies (22.7%), the participants and the doctor knew the allocation of the intervention 8,16,18,29,30. None of the studies specified whether those performing the study analysis were blinded; however, the outcomes assessed were not considered to have significantly increased the risk of bias. 

### Withdrawal and management of missing data 

In 13 studies (59.1%) the data analysis performed for all of the randomized patients does not make reference to how missing data were handled; in 9 (40.9%) studies, the data analysis excluded lost data 8, 15, 19,23,26,27,30,31,34, and in 3 (13.6%), an interim analysis was performed 8,16,17.

### Effects of the intervention

**Primary outcome (CIN).** The incidence of CIN varied between 1.67% and 17.06% for the SB side and between 1.12% and 34.48% for the 0.9% NSS side; an assessment of the percentage of patients with CIN in all the studies showed that 589 out of the 5,686 patients assessed developed CIN (10.36%); among the patients using SB, 9.03% (255/2,824) developed CIN, and among the 0.9% NSS group, 11.67% (334/2,862) developed CIN.

**Figure 3.**
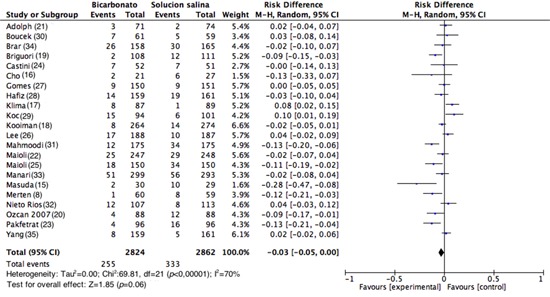
Analysis of the studies demonstrating cases of contrast-induced nephropathy. Forest plot where the number of participants and the total number of events (nephropathy contrast) in both the intervention group and the control group was observed, the point estimates of risk assessed by difference, their confidence intervals and the meta-analysis performed.

**Figure 4.**
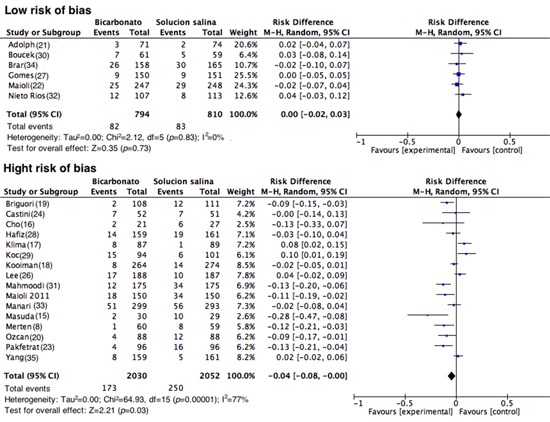
Contrast-induced nephropathy grouped according to risk of bias. Forest plot of studies grouped according to the risk of bias of the studies. Studies that were considered to have a low risk of bias included the following domains: random sequence generation, allocation concealment, and the blinding of participants and the staff that were classified as low risk or unknown risk.

In total, 22 studies were analyzed (n= 5,686). The assessment of the primary outcome indicated that the risk of developing CIN was lower among similar group that received SB; however, high heterogeneity was observed among the studies (RD= -0.03; 95% CI= -0.05 to 0.00; I^2^= 70%; *p *<0.001; Fig. 3). Nonetheless, in the analysis of the subgroup of studies with good methodological quality (generation of randomized sequence, concealment of allocation and blinding of participants and staff), 82 of 794 patients developed CIN in the SB group vs. 83 of 810 patients in the control group (RD= 0.00; 95% CI= -0.02 to 0.03; *p*= 0.83; I^2^= 0%; Fig. 4). Upon assessing the studies with a high risk of bias, the results favored the use of SB; however, heterogeneity was high (RD= -0.04; 95% CI= -0.08 to 0.00; I^2^= 77%; *p *<0.001; Fig. 4). 

### Subgroup analysis 

**Figure 5.**
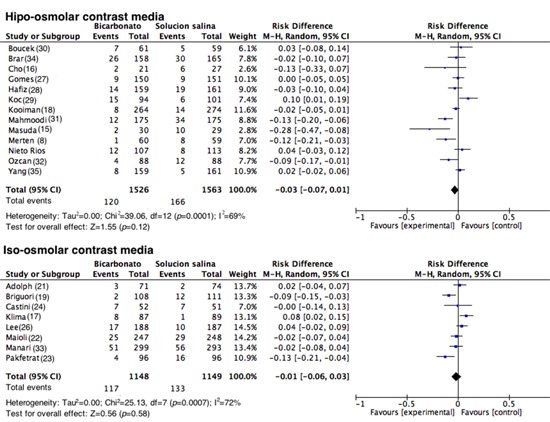
Contrast-induced nephropathy according to the contrast used. Forest plot grouping the studies according to the contrast used (iso-osmolar, hypo-osmolar). The results are expressed as risk differences with their respective confidence intervals.

**Contrast media used. **For the analysis of subgroups based on the contrast media used, the studies in which high-osmolar contrast media were applied, the group that received SB experienced 120 events out of 1,526 (7.86%) vs. 166 events out of 1,563 (10.62%) in the 0.9% NSS group (RD= -0.03; 95% CI= -0.07 to 0.01; I^2^= 69%; *p *<0.001; Fig. 5). In the studies in which iso-osmolar contrast media were used, 117 events out of 1,148 (10.2%) occurred in the SB group vs. 133 events out of 1,149 (11.50%) in the 0.9% NSS group (RD= -0.01; 95% CI= -0.06 to 0.03; I^2^= 72%; *p *<0.001; Fig. 5). One study was excluded from the analysis (26) because the type of contrast media used was unspecified. 

**Figure 6.**
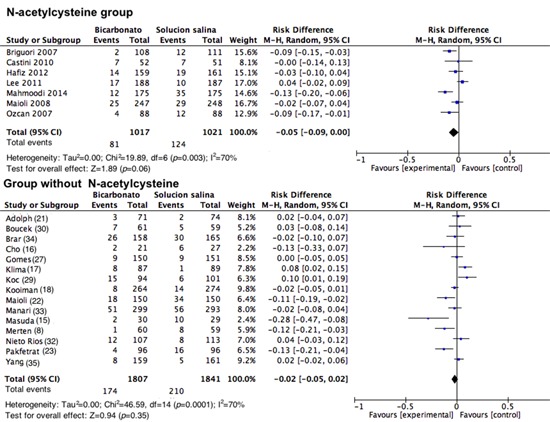
Contrast-induced nephropathy among patients grouped according to the use or non-use of N-acetyl cysteine. Forest plot grouping the studies according to the use of N-acetyl-cysteine ​​as a co-intervention with NSS or SB. The results are expressed as risk differences with their respective confidence intervals.

**Contrast-induced nephropathy in patients who received NAC. **Upon analyzing how the studies that included NAC intervention were conducted, for CIN, an RD of 0.05 was found with a 95% CI of -0.09 to 0.00 (I^2^=70%; *p *<0.001; Fig. 6). In the studies in which NAC was not used, no difference was found (RD= -0.02; 95% CI= -0.05 to 0.02; I^2^= 70%; *p *<0.001; Fig. 6). There was no analysis of low risk of bias among the studies because only one study could be included in the NAC group.

### Meta-regression 

A meta-regression analysis was performed to assess whether the quantity of contrast medium could explain the development of CIN across the primary studies. It indicated that the contrast volume did not have a statistically significant effect on the risk of CIN (*p*= 0.59). In contrast, a statistically significant relationship between diabetes and the risk of CIN was found (*p*= 0.034). Given that more than 10 studies are required to assess this variable for meta-regression, we were unable to do so with the studies with a low risk of bias because our sample included only 6 such studies. 

###  Secondary outcomes

**Figure 7.**
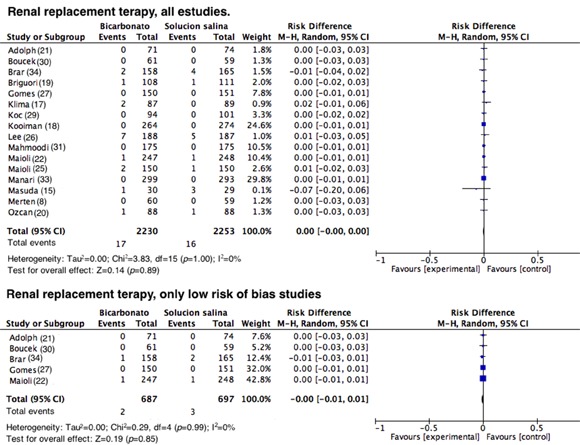
Need for renal replacement therapy in all studies and in those with a low risk of bias. Forest plot assessing the need for renal replacement therapy following the administration of contrast medium. The results are expressed as risk differences with their respective confidence intervals.

**Renal replacement therapy (RRT). **Sixteen studies reported the need for RRT after exposure to contrast media. However, such events were rare in all of the studies (RD= 0.00; 95% CI= -0.00 to 0.00; I^2^= 0%; *p*= 1; Fig. 7). This result has been previously noted and was expected given that in almost all of the studies that assessed this outcome, no events were reported. Similar results were observed when RRT was assessed in the studies that had a good methodology, with an RD= 0.00 (95% CI= -0001 to 0.01; I^2^= 0%; *p*= 0.99; Fig. 7).

**Figure 8.**
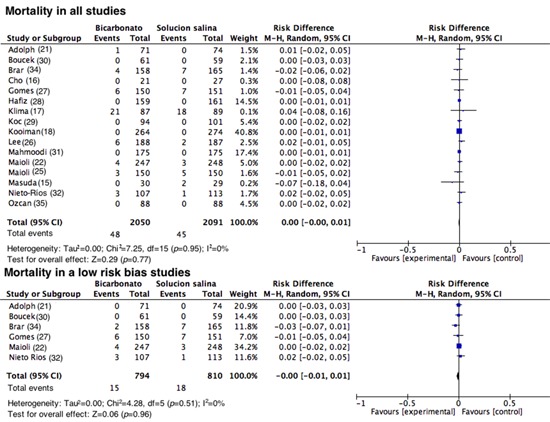
Mortality in all studies and in those with a low risk of bias. Forest plot evaluating mortality in the first 28 days after the administration of contrast media. The results are expressed as risk differences with their respective confidence intervals.

**Mortality.** Sixteen studies reported mortality among their outcomes. Similar to the outcome of RRT, this event was infrequent among the two groups (RD= 0.00; 95% CI= -0.00 to 0.01; I^2^= 0%; *p*= 0.95). When the studies with a good methodological quality were assessed, no statistically significant reduction in risk was found, with an RD= -0.00 (95% CI= -0.001 to 0.001; I^2^= 0%; *p*= 0.51; Fig. 8).

### Publication bias

**Figure 9.**
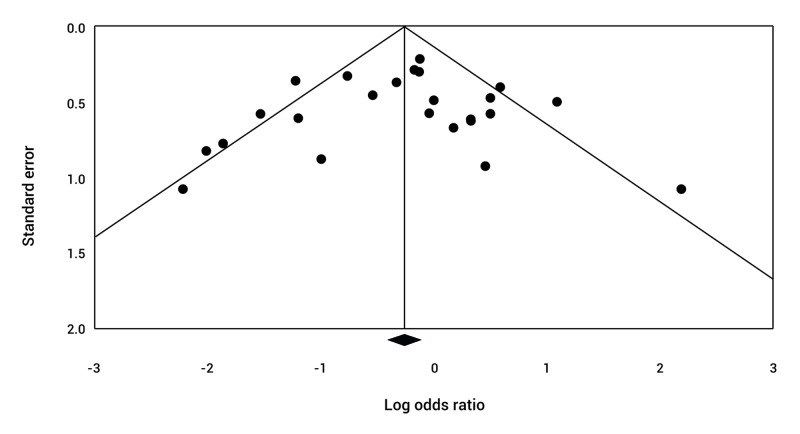
Funnel plot of all evaluated studies. Graph illustrating the dispersion of estimates of the effects of the intervention against the accuracy of each study, which increases in proportion to the size of the sample. As observed, the graph representing the studies is symmetrical, suggesting little risk of publication bias in the studies evaluated.

The funnel plot showed little asymmetry among the studies (Fig. 9). The Egger regression test generated a value of *p*= 0.69 (95%CI= -2.11 to 1.44), which indicates a low risk of publication bias among the studies. 

## Discussion 

The number of procedures that require the administration of contrast media has increased significantly in the last decade. For example, in the United States, 10 million patients per year undergo a procedure that requires contrast media 36. Moreover, approximately 658,000 persons have a percutaneous coronary intervention annually, amounting to an increase of 326% between 1987 and 2004 5. However, the use of contrast media is not without risk, and they are categorized as nephrotoxic agents.

The global rate of CIN is close to 150,000 patients per year 4. Its incidence oscillates between 0.6 to 3.0% of the general population 37 and is as high as 25.0% in high-risk patients, including those with diabetes, a history of congestive heart failure, chronic kidney disease 38 advanced age, malnutrition or concomitant use of nephrotoxic drugs (anti-inflammatory drugs, angiotensin-converting enzyme inhibitors, angiotensin II type 1 receptor antagonists and aminoglycosides) 1,38. Other risk factors reported in the literature are the volume of contrast media and the mode of administration used (arterial vs. intravenous) 1,39.

Although CIN is generally defined as a transient impairment of renal function after the administration of contrast media, it is not considered a benign complication; up to 0.8% patients may need to have temporary dialysis, and 13.0% require permanent RRT 17. Additionally, the hospital stay is prolonged and medical costs are increased, as is the risk of short- and long-term morbi-mortality 26,27. Therefore, studies that focus on strategies for preventing possible complications arising from the use of contrast media have great relevance.

Various pathophysiological mechanisms have been suggested to explain CIN. Under normal conditions, the renal medulla receives little oxygen despite having high metabolic activity for the reabsorption of substances in the S3 segment of the proximal tubules and the thick ascending limb of the loop of Henle. Consequently, mechanisms such as the release of prostaglandins, nitric oxide and adenosine that regulate renal blood flow and provide transtubular transport are required to prevent medullary hypoxia. Contrast media have direct and indirect effects on renal physiology: initially, they cause microvasculature disruption and hemodynamic changes that lead to prolonged intrarenal vasoconstriction, increased vascular resistance, decreased blood flow and osmotic diuresis, increased local oxygen consumption and induced medullary hypoxia. This effect induces the formation of reactive oxygen species that decrease the amount of nitric oxide, and the results are even more pronounced when reactive oxygen species combine with the superoxide anion to form the most powerful oxidizer, peroxynitrite, causing further damage to the endothelial cells. Increased renal vasoconstrictor activity (vasopressin, angiotensin II, dopamine, endothelin and adenosine) and reduced activity of the renal vasodilators (nitric oxide and prostaglandins) are also observed. Furthermore, the injection of contrast medium has a direct cytotoxic effect on the endothelium and renal tubular cells; it causes cell shrinkage, nuclear protrusion, fenestration of the endothelial layer, the formation of microvilli on the cell membrane and apoptosis 3,5. 

Preventive measures are the best option for all patients with risk factors for developing CIN. Different strategies have been proposed to interrupt the pathophysiology of CIN, such as the use of 1) drugs with antioxidant properties (N-acetyl cysteine [NAC], ascorbic acid, vitamin E, statins, theophylline and sodium bicarbonate), 2) vasodilators (prostaglandins, dopamine and fenoldopam), 3) alkalization (sodium bicarbonate) and 4) peri-procedural intravascular volume expansion with saline solution (NSS) 5,18,40. The usefulness of efforts to expand the intravascular space with 0.9% NSS lies in the volume, which blocks the vasoconstrictive effect of contrast on the renal medulla by suppressing the vasopressin secretion that inhibits the renin-angiotensin-aldosterone system and increases prostaglandin synthesis. In another way, the use of saline attenuates the direct toxic effects of contrast on the tubular epithelial cells by reducing the tubular reabsorption of salt and water, which allows the dilution of the intratubular fluid and the reduction of the intratubular viscosity, thus reducing the toxic effects. SB alkalinizes the liquid and reduces the rate of intratubular injury from hydroxyl radicals; thus, SB treatment is more beneficial than 0.9% NSS 18. 

Small randomized studies have shown that nephroprotection with SB initiated one hour before the administration of contrast medium can be useful for preventing CIN 41. Merten *et al*. were the first to report a significant reduction in CIN among patients hydrated with SB (1.7% vs. 13.6% *p*= 0.02); however, theirs was a single-center study with 119 patients, and it ended prematurely with no clear justification 8. Another study conducted of 7,977 patients exposed to contrast medium performed in the Rochester Mayo Clinic could not confirm Merten *et al*., initial finding regarding the protective effect of SB. In contrast, they found an increased rate of CIN in patients who received SB treatment 42. In this regard, various systematic reviews and meta-analyses have shown that SB is beneficial in preventing CIN; however, these meta-analyses showed publication bias and significant heterogeneity 11,12.

The main result of our meta-analysis, which included 22 randomized controlled clinical trials (n= 5,686 patients), suggests that the administration of SB in high-risk patients exposed to contrast media did not reduce the incidence of CIN, the need for RRT or the rate of death, compared with the use of 0.9% NSS. Additionally, no difference was found in the serum creatinine changes after the administration of contrast media.

When of all studies were analyzed, a summary effect in favor of the use of SB for CIN prevention was found, similar to the findings reported in other meta-analyses 43-46. However, many of these studies had a high risk of bias; many did not report the proper conduct and concealment of a random allocation sequence, which may lead to systematic errors within and among studies. Additionally, many of them did not blind the patients, physicians or those assessing the outcomes. Conversely, when only the studies with a low risk of bias were analyzed, the protective effect of SB disappeared, as did the heterogeneity (RD= 0.00; 95% CI= -0.02 to 0.03; *p*= 0.83; I^2^= 0%). 

Another objective of this study was to evaluate whether the type of contrast used could influence the potential nephroprotective effect of SB. Subgroup analysis showed no significant differences among the patients who received hypo-osmolar contrast (RD= -0.03; 95% CI= -0.07 to -0.01; I2= 69%; *p* <0.001) and those who received iso-osmolar contrast (DR= -0.01; 95% CI= -0.06 to -0.03; I2= 72%, *p* <0.001). This finding is consistent with recent studies that have also failed to show a significant difference in the incidence of CIN after the administration of iso-osmolar media vs. low-osmolarity media 38,47. 

The meta-regression analysis aimed to assess whether the volume of contrast used was related to the potential protective effect of SB against the development of CIN. In the studies that reported this variable, it was not possible to establish a direct relationship with SB, while the literature indicated that the volume of contrast used increases the risk of CIN 48; however, a proviso must be made that some of these studies did not use the best methodological standards. The association between a history of diabetes mellitus and the risk of CIN was also evaluated; a statistically significant relationship was found (*p*= 0.034), indicating that diabetes is a risk factor. 

In this study, secondary outcomes, such as death, the need for RRT and changes in the creatinine level, showed no improvement with SB use compared with 0.9% NSS use. This may be related to the small number of subjects included in the tests, the design methodology, the insufficient power to detect these differences and the short-term monitoring used. Even after the outcomes were analyzed according to methodological quality, the results did not change.

### Study limitations

The major limitation found in this meta-analysis was the poor methodological quality of many of the studies included, which is related to problems of randomization, concealment and blinding. These aspects negatively influenced the estimated effect of different outcomes. 

Regarding the inclusion criteria, the definition of chronic kidney disease was very heterogeneous and despite being based on the creatinine value and/or GFR. The range of cutoff points for these variables was very wide and did not take gender, age and body mass into account. Most of the trials included in our study used the elevation of creatinine within 48 h after exposure to contrast medium as the definition of CIN, without considering that the elevation of serum creatinine may occur 4 to 5 days after exposure and therefore the effect of hydration protocols cannot be estimated well.

Another important limitation is the lack of uniformity in the dose and duration of therapy with SB or 0.9% NSS among the different clinical trials. Likewise, the average volume of contrast medium was variable, and none of the studies reported the patients' weights to allow an estimation of the dose per kilogram of body weight. Finally, we believe that these results cannot be generalized, and it must be remembered that the patients included were usually undergoing cardiac procedures. Furthermore, the sample size would not allow a sufficient power, and the monitoring periods of the studies were excessively short.

## Conclusions

This meta-analysis of clinical trials showed that the use of SB is not superior to the use of 0.9% NSS, alone or with concomitant use of NAC, to prevent CIN among patients who are exposed to contrast media and have risk factors for CKD. Furthermore, there is no evidence to suggest that either intervention has greater beneficial effects in terms of reducing mortality and the need for RRT. These results should be considered in the context of the marked heterogeneity among the different trials. Thus, further studies with higher power and better standards and protocols are required to allow a meta-analysis of studies with a low risk of bias that can help to determine what the ideal intervention is for preventing CIN.
